# Development and early qualitative evidence of two novel patient-reported outcome instruments to assess daily functioning in people with early-stage Parkinson’s

**DOI:** 10.1186/s41687-023-00577-9

**Published:** 2023-04-20

**Authors:** Thomas Morel, Sophie Cleanthous, John Andrejack, Roger A. Barker, Milton Biagioni, Geraldine Blavat, Bastiaan R. Bloem, Babak Boroojerdi, William Brooks, Paul Burns, Stefan Cano, Casey Gallagher, Lesley Gosden, Carroll Siu, Ashley F. Slagle, Natasha Ratcliffe, Karlin Schroeder

**Affiliations:** 1 UCB Pharma, Allée de la Recherche 60, 1070 Anderlecht, Brussels, Belgium; 2 Modus Outcomes, a Division of Thread, London, UK; 3 Parkinson’s Foundation, New York, NY USA; 4 University of Cambridge, Cambridge, UK; 5 Radboud University Medical Centre; Donders Institute for Brain, Cognition and Behaviour; Department of Neurology; Centre of Expertise for Parkinson & Movement Disorders, Nijmegen, The Netherlands; 6 UCB Biosciences GmbH, Monheim, Germany; 7 Parkinson’s UK, London, UK; 8 Aspen Consulting, LLC, Steamboat Springs, CO USA

**Keywords:** Parkinson’s, Early-stage Parkinson’s, Patient expert, Patient-reported outcome instrument, Clinical outcome assessments, Qualitative evidence, Slowness, Mobility

## Abstract

**Background:**

Previous research on concepts that are important to people living with early-stage Parkinson’s indicated that ‘*functional*’ slowness, fine motor skills, and subtle gait abnormalities are cardinal concepts that are not comprehensively captured by existing patient-reported outcome (PRO) instruments that are used in clinical practice and research to assess symptoms and daily functioning within this patient population. We sought to develop novel PRO instruments to address this unmet need.

**Methods:**

PRO instrument development was led by a multidisciplinary research group, including people living with Parkinson’s (termed ‘patient experts’), as well as patient engagement and involvement, regulatory science, clinical, and outcome measurement experts. A first set of PRO instruments, termed Early Parkinson’s Function Slowness (42 items) and Early Parkinson’s Mobility (26 items), were drafted to capture ‘*functional*’ slowness, fine motor skills, and subtle gait abnormalities. These PRO instruments were used in cognitive debriefing interviews with people living with early-stage Parkinson’s (who were not involved with the multidisciplinary research group) to identify issues with relevance, clarity, ease of completion, conceptual overlap, or missing concepts.

**Results:**

Sixty people living with early-stage Parkinson’s were interviewed, which led to refining the items to 45 for the Early Parkinson’s Functional Slowness and 23 for the Early Parkinson’s Mobility PRO instruments. Refinement included rewording items to address clarity issues, merging or splitting items to address overlap issues, and adding new items to address missing concepts. The Early Parkinson’s Function Slowness PRO instrument resulted in a multidimensional instrument covering upper limb, complex/whole body, general activity, and cognitive functional slowness. The Early Parkinson’s Mobility PRO instrument resulted in comprehensive coverage of everyday mobility tasks, with a focus on gait concepts, plus complex/whole body, balance, and lower limb mobility.

**Conclusions:**

The Early Parkinson’s Function Slowness and Early Parkinson’s Mobility PRO instruments aim to address gaps in existing PRO instruments to measure meaningful symptoms and daily functioning in people living with early-stage Parkinson’s. Utilizing a meticulous study design led by a multidisciplinary research group that included patient experts helped to ensure that the PRO instruments were patient-centric, content valid, and meaningful from a clinical and measurement perspective.

**Supplementary Information:**

The online version contains supplementary material available at 10.1186/s41687-023-00577-9.

## Abstract (highlights)

Clinical outcome assessment tools can be used to show how a disease impacts a person’s daily life. These outcomes are assessed with tools like surveys or questionnaires. In Parkinson’s, most of the tools currently available do not reflect the things that are important to people living with early-stage Parkinson’s. Instead, these tools were designed to assess people in later stages of the disease.

In this study, the research team created two new tools to assess daily functioning in early-stage Parkinson’s. People with early-stage Parkinson’s were interviewed to test the new tools. The language and questions in the tools were changed based on feedback from the interviews. The tools were reviewed by a large team, including patient experts and healthcare professionals involved in Parkinson’s care. Experts in outcome measures and clinical trials also reviewed the tools. The review step ensured that the tools are easy to understand and appropriate for people living with early-stage Parkinson’s.

The new tools focus on the symptoms and experiences that are most important to people living with early-stage Parkinson’s. We hope that the tools will more clearly show the impact of early-stage Parkinson’s and how it progresses. Further testing of these new tools is needed. The aim is to use them in future clinical trials to measure the experience of early-stage Parkinson’s more accurately.

**Supplementary Information:**

The online version contains supplementary material available at 10.1186/s41687-023-00577-9.

## Background

Parkinson's is a neurological disease affecting populations with fast-growing prevalence, disability, and death [1]. The onset of subtle, yet important, motor (such as, bradykinesia) and non-motor (such as, constipation, rapid eye movement sleep behaviour disorder, and hyposmia) symptoms commonly start years before diagnosis [2–4]. These prodromal manifestations are followed by the more characteristic motor symptoms (such as, tremor, rigidity, and postural problems, as well as bradykinesia) that lead to clinical diagnosis [3, 4]. Throughout the prodromal phase and into early-stage Parkinson’s, the disease burden of Parkinson’s and its impact on people living with Parkinson’s is relatively under-recognized. Currently, there is no widely accepted definition of early-stage Parkinson’s among the scientific and regulatory communities; it can be defined by time since formal diagnosis (for example, less than 5 years), functional impairment (assessed by the Hoehn and Yahr scale), or a combination of both [5–8]. In mid- and late-stage Parkinson’s, physical disability becomes more evident, and non-motor symptoms such as cognitive impairment, sleep disturbances, and autonomic dysfunction become very prevalent [3, 4]; the combination of these motor and non-motor features negatively impact quality of life and can cause loss of independence [9–11].

A therapy that slows or stops Parkinson’s progression and prevents disability remains the single most important unmet need in the treatment of Parkinson’s [4, 12]. Although major scientific advances have identified novel targets and promising drug candidates, there are no validated biomarkers for Parkinson’s and the number of failed attempts to develop therapies that slow disease progression and prevent decline in functional ability continue to grow [12–15]. While lack of efficacy or inadequacy of study designs may explain some of the disappointing results, the clinical outcome assessments (COAs) used have been developed largely for people living with later stages of Parkinson’s, and evidence suggests that they are not fit for purpose in the context of early-stage Parkinson’s [16–19]. For example, these COAs are especially limited in assessing the progression of functional impairment in early-stage Parkinson’s [20].

Developing fit-for-purpose COAs requires a thorough empirical exploration of the concepts of interest in the target context of use [21]. While the development of patient-reported outcome (PRO) instruments would benefit from patient involvement, it is not currently standard practice [22]. Recent guidance from the US Food and Drug Administration supports patient-focused drug development and describes how stakeholders (patients, researchers, medical product developers, and others) can collect and submit patient experience data, and other relevant information from patients and caregivers for medical product development and regulatory decision-making [23–25]. This guidance addresses patient involvement in COA development, highlighting the increasing importance regulatory bodies place on the role of patients in research and product development [26].

**Fig. 1:**
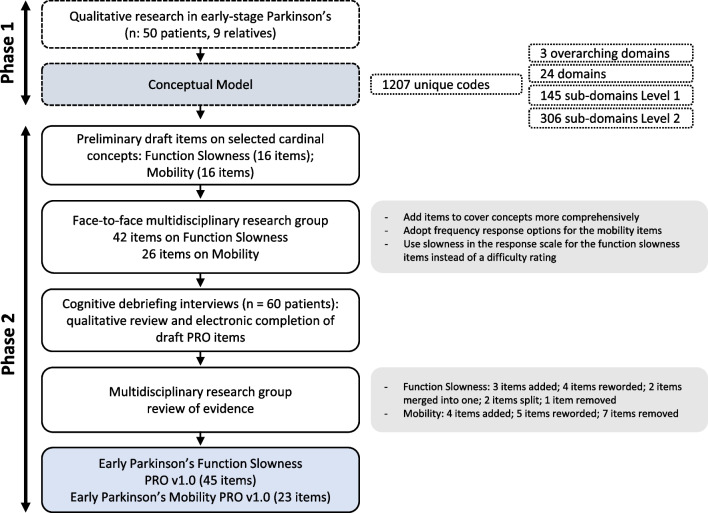
An overview of the research and development process across phases 1 [17, 19] and 2. PRO, patient-reported outcome

UCB Pharma, Parkinson’s UK and Parkinson’s Foundation set up a multidisciplinary research group to co-develop a patient-focused outcome measurement strategy in early-stage Parkinson’s, geared to improve the ability to assess meaningful changes in daily functioning in early-stage Parkinson’s. A two-phase approach was adopted (Fig. 1) [17, 19]; the first phase included eliciting the symptoms and impacts important to people living with early-stage Parkinson’s [19]. It specifically identified concepts that are ‘cardinal’ within the early-stage Parkinson’s experience, which was informed by the relative frequency of reported concepts, as well as feedback from patient experts and movement disorder specialists [19]. The concepts identified were ‘*functional’* slowness, tremor, rigidity/stiffness, fine motor skills, subtle gait abnormalities, fatigue, depression, sleep/dreams, and pain.

After reviewing the qualitative findings from the first phase of the study, the multidisciplinary research group concluded that functional slowness (where people with Parkinson’s are starting to be slower at completing tasks, but have not yet encountered significant difficulties), fine motor skills, and subtle gait abnormalities would be the most relevant concepts to include in outcome assessments for people living with early-stage Parkinson’s [19]. It was hypothesized that these concepts should be measured as they combine patient relevance (i.e., identified as cardinal concepts in the earlier disease conceptual model), clinical meaningfulness, a potential to improve with treatment, and an ability to translate into endpoints capable of capturing change in a study setting within a maximum time window of 24 months.

During the first phase, a literature review (further information available upon request) identified 15 legacy PRO instruments including the 39-item Parkinson’s disease questionnaire (PDQ-39) and MDS-UPDRS part I and II. Preliminary concept-to-item mapping of these legacy PRO instruments was conducted; it was uncovered that none had comprehensive coverage of the lived-experience of early-stage Parkinson’s and, as such, are of limited use in this setting [17]. Analysis of the literature review findings led the multidisciplinary research group to conclude that novel PRO item generation was warranted to accurately reflect living with early-stage Parkinson’s by quantifying functional slowness, fine motor skills, and subtle gait abnormalities.

Here, in phase 2, we report the outcome of this process, namely the development of the Early Parkinson’s Function Slowness and Early Parkinson’s Mobility PRO instruments.

## Methods

### Aim

We aimed to develop novel PRO instruments that accurately reflect the lived experience of early-stage Parkinson’s for use in future clinical trials involving people with the condition.

### Multidisciplinary research group and cognitive debriefing participants

PRO item development, study design, interpretation of findings, and decision-making on PRO development and future evidence needs were led by a multidisciplinary research group. The multidisciplinary research group comprised six people living with Parkinson’s (termed ‘patient experts’ who acted as patient researchers and patient reviewers [27]), patient engagement and involvement experts from patient advocacy organizations (PAOs) (Parkinson’s UK [n = 1] and Parkinson’s Foundation, USA [n = 2]), a regulatory science expert (n = 1), outcome measurement experts (n = 2), clinical experts (i.e., trained neurologists in movement disorders [n = 3]), and sponsor representatives who specialized in clinical development (n = 2). Using their research support networks, the PAOs selected patient experts that reflected diversity in gender, educational background, geographical location, time since diagnosis, and past involvement in clinical studies.

Participants, who were the subject of the cognitive debriefing interviews, were termed ‘cognitive debriefing participants’ and acted as patient discussants [27]. They were recruited through the PAOs and were separate to the patient experts involved in the multidisciplinary research group. Cognitive debriefing participants included study participants from phase 1, as well as new participants. In the UK, an invite was shared with the members of the Parkinson’s UK Research Support Network by email. In the USA, the Parkinson’s Foundation aimed to recruit people diagnosed within the past two years and so, shared a targeted invitation to members of its Research Advocacy Program (members are required to report their year of diagnosis) and Newly Diagnosed Initiative (members had just received a diagnosis from a physician). After providing written informed consent, all cognitive debriefing participants completed an electronic eligibility screening form.

Cognitive debriefing participants were people living with early-stage Parkinson’s, with a target enrollment of 4:1 unilateral (self-reported to be confined to one side of the body) or bilateral Parkinson’s motor manifestations. Exclusion criteria included heart and circulatory problems, kidney disease, type 1 or type 2 diabetes, inflammatory gastrointestinal disorders, hepatitis B or C virus, and HIV. Completion of the eligibility screening form enabled identification of cognitive debriefing participants meeting the inclusion criteria; the form was developed with clinical and patient experts to ensure ease of understanding.

### PRO item development

Initially, a set of 16 draft items focusing on functional slowness and 16 focusing on mobility were developed by the patient experts and experts in clinical outcome measures. These items were generated from quotes and wording originating from interview responses in phase 1 [19] in line with good research practices [28]. Each item was constructed to target a single concept, and the number and wording of response options were purposefully considered to reflect variation in the concept being measured.

In October 2019, the multidisciplinary research group held a face-to-face meeting to further develop and revise the preliminary version of the item sets, including the terminology used. In three rounds of review, the multidisciplinary research group provided feedback and reviewed the items for comprehensiveness against the conceptual model and decided to: develop additional items to cover functional slowness and mobility concepts more comprehensively; include cognitive slowness items, as both motor and cognitive aspects contributed to some features of functional slowness (most specifically when a person living with early-stage Parkinson’s needed to react to something); implement a frequency-based response scale for mobility items to capture the fluctuation in experience of a person living with Parkinson’s; and base response scales for the functional slowness items on levels of slowness rather than difficulty rating, as patient experts stressed that the time taken to perform a daily functioning task is more meaningful than the difficulty of the task. Insights from the patient experts were used to refine items over and above those generated directly from the interview transcripts to ensure that the wording reflected the perspective of the person living with Parkinson’s and that the context was clear.

An item tracking matrix was used to document the evolution of items and justifications behind the decisions for all versions of the instruments.

The draft Early Parkinson’s Function Slowness item set was revised to include 42 items scored on a 5-point Likert scale ranging from ‘not at all’ to ‘extremely slow’ within a 7-day recall period. The draft Early Parkinson’s Mobility item set was revised to include 26 items and was scored on a 5-point Likert scale ranging from ‘not at all’ to ‘extremely’ within a 7-day recall period. Both draft item sets were used for cognitive debriefing interviews. The response option structure for both item sets was aimed to allow granular and marginal assessment of change over time.

### Cognitive debriefing interviews

Cognitive debriefing participants, in their role as patient discussants [27], were emailed a copy of the refined PRO item sets using a secure online survey website (REDCap). One-on-one semi-structured cognitive debriefing interviews (lasting 60 to 90 min) were then conducted via telephone by four research personnel from Modus Outcomes who introduced themselves, the goals of the interview, processes, and procedures. Cognitive debriefing participants were assigned to either Group A or Group B alternately as they enrolled in the study, and a separate interview guide was developed for each group. Both groups reviewed all items; however, Group A provided a full in-depth, item by item verbal review of the Early Parkinson’s Function Slowness PRO instrument and Group B did the same for the Early Parkinson’s Mobility PRO instrument, which ensured all PRO instruments were covered in the allocated time period. No repeat interviews were conducted.

During the interviews, cognitive debriefing participants were asked to complete the PRO item sets online and were encouraged to ‘think aloud’ to elicit spontaneous responses. Interviewers also asked cognitive debriefing participants open-ended questions to encourage them to reveal any difficulty or ambiguity in comprehending the items and/or choosing a response option, to comment on the relevance of items, as well as any overlap between items, and to flag missing concepts important to them. Examples of the different questions used in the interviews are shown in Additional file 1: Table S1. All feedback was collected in real-time during the interviews.

### Qualitative analysis

Interviews were recorded and the audio files were transcribed verbatim. No copies of the transcript were shared with the cognitive debriefing participants for comment/feedback. ATLAS.ti software was used to code the transcripts, which were then analyzed thematically [29] using detailed open line-by-line inductive coding [30, 31]. The themes were derived inductively from the interviews. The transcripts were coded by four research personnel from Modus Outcomes using a coding guide, which provided principles and a formatting framework for the open coding. Parallel coding was completed by two researchers for the first two transcripts; the research team reviewed these codes to ensure alignment before completing the remaining coding. Researchers were not permitted to code the transcripts for the interviews they conducted. The research team had regular meetings to discuss coding results and adjust coding style if required.

This qualitative analysis was used to highlight the issues reported by cognitive debriefing participants with the new PRO items in relation to relevance (including identifying items that were least or most relevant to early-stage Parkinson’s), clarity, conceptual overlap, and missing concepts.

### Multidisciplinary research group review

During three rounds of reviewing the qualitative findings, which included two teleconferences (separate to the previous face-to-face meeting), the multidisciplinary research group refined the PRO instrument content, language, structure, and instructions according to four criteria: (i) comprehensiveness and relevance of the item: the item set should cover all concepts that are included in the conceptual model and those important to people living with early-stage Parkinson’s, as well as no relevance issues being identified [32]; (ii) conceptual uniqueness: items should reflect different aspects/levels of a unique concept [32]; (iii) item clarity: the items should be easily understood with no consistent issues with ambiguity/misinterpretation [32]; and (iv) appropriateness of response scale: the scale should be easy to use with no consistent issues identified with choosing a response [32].

## Results

### Sample

A total of 60 people living with early-stage Parkinson’s (n = 30 recruited by both Parkinson’s UK and Parkinson’s Foundation) completed the cognitive debriefing interviews in this study. The 60 cognitive debriefing participants had either completed phase 1 of the research (n = 43) or were new to the research (n = 17); these participants were assigned to Group A (n = 31) or Group B (n = 29).

Most cognitive debriefing participants had unilateral manifestations (n = 42/60, 70%). The median time since diagnosis was 1 year in the USA and 4 years in the UK due to the different study recruitment approaches used by the two PAOs. Most cognitive debriefing participants were either unemployed or retired (n = 40, 67%) and 25% of these participants lived alone (n = 15). There were some differences between the UK and USA cognitive debriefing participants, including gender split, unilateral or bilateral manifestations, and years since formal diagnosis. Further details of cognitive debriefing participants, including population differences between the UK and USA, are presented in Table 1.

**Table 1:** Cognitive debriefing participant characteristics

		Participant sample	
		UK (N = 30)	USA (N = 30)
Age (years)	Mean (SD)	64.1 (10.3)	64.1 (8.3)
	Range	37–84	45–75
Gender, n (%)	Female	19 (63)	15 (50)
	Male	11 (37)	15 (50)
Type, n (%)	Unilateral	24 (80)	18 (60)
	Bilateral	6 (20)	12 (40)
Year(s) since formal diagnosis, n^*^	Mean (SD)	4.9 (3.7)	2.0 (0.8)
	Range	1–17	1–3
	Median	4	1
	Less than 2 years, n (%)	7 (23)	20 (67)
	More than 2 years, n (%)	23 (77)	8 (27)
Ethnicity, n (%)	White	30 (100)	27 (90)
	Black	–	2 (7)
	Asian	–	1 (3)
Education, n (%)^*^	PhD/DPhil	1 (3.5)	–
	Postgraduate degree	5 (17)	12 (40)
	Bachelor/Undergraduate/Associate degree	9 (31)	10 (33)
	Pre-higher education^†^	12 (40)	8 (27)
	Other	2 (7)	–
Employment status, n (%)	Working full-time	5 (16)	8 (27)
	Working part-time	2 (7)	5 (17)
	Unemployed	2 (7)	4 (13)
	Retired	21 (70)	13 (43)
Living situation, n (%)	Living alone	8 (26)	7 (23)
	Living as couple	20 (67)	14 (47)
	Living with family	2 (7)	9 (30)

A-Level: Advanced Level; DPhil: Doctor of Philosophy; GCSE: General Certificate of Secondary Education; GED: General Educational Development; HNC: Higher National Certificate; NVQ: National Vocational Qualification; O-Level: Ordinary Level; PhD: Doctor of Philosophy; SD, standard deviation; UK, United Kingdom; USA, United States of America

*This table presents sample characteristics of available data. The data for year(s) since formal diagnosis were missing for two cognitive debriefing participants from the USA and data for the education status of one cognitive debriefing participant from the UK was missing

^†^Pre-higher education includes: GCSE/O-Level equivalent; A-Level/Level 3 NVQ or equivalent; and HNC/Level 4 NVQ or equivalent for UK cognitive debriefing participants, and high school graduate/GED equivalent and some college for USA cognitive debriefing participants

### Refinement of the draft Early Parkinson’s Function Slowness PRO

In general, cognitive debriefing participant feedback strongly endorsed the relevance of the item content of the draft Early Parkinson’s Function Slowness PRO instrument (Table 2) to their experience of living with early-stage Parkinson’s. Almost all items (40/42) were flagged by at least one cognitive debriefing participant as being the most relevant to them, and no patterns of items were identified as not relevant. A small number of items were identified as potentially ambiguous including ‘Lifting or carrying things’ (n = 4, 13.3%), ‘Multitasking’ (n = 6, 20%), ‘Keeping things organized’ (n = 4, 13.3%), and ‘Reacting to things in real time to avoid potential accidents’ (n = 7, 23.3%).

**Table 2:** Cognitive debriefing participant feedback on the Early Parkinson’s Function Slowness PRO instrument

Item name	Early Parkinson’s Function Slowness PRO				
	Group A and B* N = 59		Group A^†^ N = 30		
	Most relevant n	Less/least relevant n	Not relevant n	Clarity issue^‡^ n	Response problem n
Handwriting	13	1	1	1	3
Typing	4	1	–	1	1
Using a computer mouse	7	1	1	–	3
Doing up buttons	5	2	–	2	1
Doing up a zip	1	2	1	**3**	–
Brushing your teeth	5	4	–	1	1
Opening jars, or bottles	3	1	1	–	5
Using a knife	1	2	2	1	1
Using a fork	1	2	2	1	3
Getting a card or money in or out of a wallet	9	1	–	1	1
Counting money	5	3	2	1	1
Using a smart phone or tablet	3	–	1	2	2
Opening envelopes	1	2	1	–	–
Taking a plug in or out of a socket	–	1	–	–	–
Accessing or using your pockets	5	–	–	1	–
Buckling a seat belt	1	–	–	1	3
Reaching for things high up on a shelf	1	5	1	–	1
Carrying or moving things	2	1	1	1	2
Lifting or carrying things	3	1	1	**4**	1
Walking inside the house	1	3	1	–	2
Climbing up or down the stairs	3	3	1	–	4
Walking on uneven ground	5	2	–	–	2
Walking on a busy street or crowded area	2	1	–	–	2
Getting in or out of a chair	2	2	–	1	–
Getting in or out of bed	3	1	1	–	–
Putting on shoes	3	1	1	1	1
Dressing	6	4	2	2	–
Having a shower	2	3	1	–	–
Preparing food or cooking	5	1	2	–	1
Eating	4	–	1	2	1
Performing household chores	2	1	1	1	–
Running errands outside the house	–	1	1	1	1
Doing your hobbies or other leisure activities	4	2	1	2	–
Performing your work or tasks within your daily routine	1	–	1	2	–
Multitasking	4	–	1	**6**	2
Keeping things organized	1	1	–	**4**	2
Finding the right word to say in the middle of a conversation	6	–	–	1	3
Speaking	3	–	1	–	3
Thinking or processing things	2	–	1	–	–
Reading	1	2	1	–	1
Following written instructions	1	1	1	–	–
Reacting to things in real time to avoid potential accidents	1	–	1	**7**	–

PRO: patient-reported outcome

*Group A, N = 30; Group B, N = 29. One transcript from Group A was unable to be analysed due to poor audio quality, resulting in 30, rather than 31, cognitive debriefing participants for this group

^†^No issues were identified within the recall period

^‡^Items with greatest clarity issues are marked in bold

Within the draft Early Parkinson’s Function Slowness PRO instrument, conceptual overlap was reported more frequently by cognitive debriefing participants for the following items: ‘Doing up buttons/Doing up a zip’ (n = 3), ‘Using a knife/Using a fork’ (n = 5), and ‘Carrying or moving things/Lifting or carrying things’ (n = 10). No issues with the length of the 7-day recall period were mentioned. Three new items were also added to cover concepts suggested by cognitive debriefing participants as relevant but missing from the instrument: ‘Retrieving coins from a wallet or purse’, ‘Picking a coin off a table’, and ‘Tying shoelaces’. Cognitive debriefing participants indicated some issues with the response scale for 28 items, with ‘Opening jars, or bottles’ having the most mentions (n = 5, 16.7%), relating to the fact that issues other than slowness might cause individuals to be slow with performing these activities, such as stiffness or fine motor skills; in other words, pointing out their perceived cause of the slowness. However, no action was taken as the cause of slowness was not relevant in the shaping of the PRO instrument and the issues identified did not indicate a problem with selecting one of the available response options. Any changes made to the Early Parkinson’s Function Slowness PRO are included in Table 3 and feedback on the items that formed this PRO instrument are included in Table 2 and Additional file 1: Table S2.

**Table 3:** Refinement of PROs

PRO scale or item (version 0.3 as presented at the CD interviews)	Action and reason for decision*	Revised items (if applicable) as included in final version 1.0
Early Parkinson’s Function Slowness PRO*	Consensus decision to add 3 items based on PE insights and PI evidence	Picking a coin up off a table Retrieving coins from a wallet or purse Tying shoelaces
Doing up buttons	Keep and explore further at next stage of validation since PE endorsed the relevance of the item	No changes made
Doing up a zip	Item reworded for USA English appropriateness based on PE insights	Doing up a zipper
Using a knife		
	Consensus decision to merge the two items due to conceptual overlap	Using a knife and fork
Using a fork		
Counting money	Consensus decision to reword item to reduce ambiguity; based on PE insights that also addressed UK and US cultural/language difference	Counting paper money
Carrying or moving things	Item split into two to reflect two unique concepts and reworded to focus on heavy objects; based on PE insights and consensus	Carrying heavy objects Moving heavy objects
Lifting or carrying things	Decision to remove based on PE insights as ‘lifting’ had no additional conceptual relevance to carrying	Item removed
Climbing up or down the stairs	Consensus decision to reword to more appropriate terminology	Walking up or down the stairs
Walking on uneven ground		
	Decision to keep and explore further at next stage of validation since PE endorsed the unique conceptual relevance and distinct value of both items	No changes made
Walking on a busy street or crowded area		
Doing your hobbies or other leisure activities		
	Consensus decision to retain wording and explore at next stage of validation. These items relate to important and relevant concepts, but further specification may limit generalizability at this stage	No changes made
Performing your work or tasks within your daily routine		
Multitasking	Item reworded to reduce ambiguity on the basis of PE insights	Doing more than one task at the same time
Thinking or processing things	Consensus decision to retain wording and explore at next stage of validation	No changes made
Reacting to things in real time to avoid potential accidents	Based on PE insights, this item was split to reduce ambiguity and differentiate between the situation of inside and outside the home	Reacting in real time to avoid potential hazards inside the home Reacting in real time to avoid potential hazards outside the home
Early Parkinson’s Mobility*	Consensus decision to add 4 items based on PE insights and PI evidence	Difficulty turning on the spot Difficulty getting out of a car Difficulty changing direction whilst walking Difficulty walking in low lighting
Difficulty getting out of a chair	Consensus decision to reword these items to reduce ambiguity and more accurately reflect the experience of living with early-stage Parkinson’s; based on PE insights that also addressed UK and US cultural/language differences	Difficulty standing up from sitting
Difficulty walking outdoors		Difficulty walking on uneven ground
Difficulty walking in unfamiliar places		Difficulty walking on unfamiliar ground
Difficulty bending		Difficulty bending to pick up something from the floor
Needing to put more effort on your walking	Consensus to include ‘into your walking’ rather than ‘on your walking’ to make the item more grammatically correct	Needing to put more physical effort into your walking
Difficulty rolling over in bed	Consensus decision to keep and explore further at next stage of validation since the concepts were deemed relevant by PI and PE	No changes made
Needing to concentrate on your walking		
Clumsiness when walking		
Shuffling when walking		
Problems with your balance when moving	Consensus to remove item due to conceptual overlap with the ‘balance when walking’ item	Item removed
Problems with your balance when walking	Consensus to keep since overlapping item ‘problems with your balance when moving’ was removed	No changes made
Difficulty standing for long periods	Consensus decision to remove items on the basis of a problematic measurement framework around the variable interpretation of ‘long time’ between responders	Items removed
Difficulty sitting for long periods		
Difficulty walking for a long time		
Problems with your arm swing when walking	Consensus decision to remove as item relates to functional slowness rather than difficulty with mobility	Item removed
Sudden freezing when walking		
	Consensus decision to remove as both items do not sufficiently relate to the cardinal aspects of mobility issues in early-stage Parkinson’s	Items removed
Falling		

*Decision/action based on (i) evidence from interviews with people living with early-stage Parkinson’s; (ii) patient expert insights; and (iii) consensus amongst the multidisciplinary research group

CD: cognitive debriefing; PE: patient experts; PI: patient interviews; PRO: patient-reported outcome; UK: United Kingdom; USA: United States of America

Four different themes for functional slowness items were identified by the multidisciplinary research group, which included the contribution and endorsement of patient experts; as such, the 45 items in the final draft Early Parkinson’s Function Slowness PRO were provisionally grouped into the following domains: upper limb function slowness (19 items); complex/whole body function slowness (9 items); activities slowness (9 items); and cognitive function slowness (8 items).

### Refinement of the draft Early Parkinson’s Mobility PRO

Almost all draft Early Parkinson’s Mobility PRO instrument items (23/26) were flagged by at least one cognitive debriefing participant as being the most relevant to them and all items were of some relevance to cognitive debriefing participants (Table 4). The following items were identified as the most ambiguous: ‘Difficulty getting out of a chair’ (n = 4, 6.8%), ‘Difficulty bending’ (n = 4, 6.8%), ‘Difficulty sitting for long periods’ (n = 6, 10.2%), ‘Difficulty walking for a long time’ (n = 4, 6.8%), ‘Difficulty walking outdoors’ (n = 5, 8.5%), ‘Difficulty walking in unfamiliar places’ (n = 6, 10.2%), ‘Needing to put more effort on your walking’ (n = 5, 8.5%), and ‘Problems with your balance when moving’ (n = 5, 8.5%).

Within the Early Parkinson’s Mobility PRO instrument, conceptual overlap was reported more frequently by cognitive debriefing participants for the following items: ‘Needing to concentrate on your walking/Needing to put more effort on your walking’ (n = 3), ‘Problems with your balance when walking/Problems with your balance when moving’ (n = 5), and ‘Clumsiness when walking/Shuffling when walking’ (n = 3). Four items were added to the Early Parkinson’s Mobility PRO to address conceptual gaps identified in the interviews when cognitive debriefing participants provided feedback on ‘missing concepts’. These included ‘Difficulty walking in low lighting’, ‘Difficulty changing direction whilst walking’, ‘Difficulty turning on the spot’, and ‘Difficulty getting out of a car’.

A total of 17 out of 26 Early Parkinson’s Mobility PRO items were reported as having a response problem by a small number of cognitive debriefing participants, with the highest being Item 14 ‘Dragging your leg or foot when walking’ (n = 4, 13.8%). No consistent issues were identified to warrant adjustment of the response scale. An issue with the length of recall period was reported for six items: ‘Difficulty getting out of a chair’, ‘Difficulty walking for a long time’, ‘Dragging your leg or foot when walking’, ‘Needing to concentrate on your walking’, ‘Needing to put more effort on your walking’, and ‘Falling’; however, a consensus was made to retain the commonly used 7-day recall period as these PRO instruments are intended to be used in clinical trials and no issues with the recall period were identified for the majority of items. After deliberation, the multidisciplinary research group decided that removing items relating mobility to functional status, rather than difficulty, from the Early Parkinson’s Mobility PRO would better reflect the everyday experience of early-stage Parkinson’s, as well as reducing conceptual overlap and aiding understanding. As such, the following items were removed: ‘Difficulty twisting in seated position’, ‘Falling’, ‘Sudden freezing when walking’, ‘Problems with your balance when moving’, ‘Problems with your arm swing when walking’, ‘Difficulty walking for a long time’, ‘Difficulty standing for long periods’, and ‘Difficulty sitting for long periods’.

Any changes made to the Early Parkinson’s Mobility PRO are included in Table 3 and feedback on the items that formed this PRO instrument are included in Table 4 and Additional file 1: Table S2. The final draft Early Parkinson’s Mobility PRO instrument consists of 23 items covering issues with everyday mobility tasks, with a focus on gait concepts, plus complex/whole body, balance, and lower limb mobility.

**Table 4:** Cognitive debriefing participant feedback on the Early Parkinson’s Mobility PRO instrument

Item Name	Early Parkinson’s Mobility PRO				
	Group A and B* N = 59		Group B N = 29		
	Most relevant^†^ n	Not relevant n	Clarity issue^‡^ n	Response problem n	Recall period n
Difficulty getting out of a chair	4	2	**4**	3	2
Difficulty sitting from standing up	1	2	1	1	–
Difficulty bending	3	2	**4**	–	–
Difficulty sitting for long periods	7	3	**6**	1	–
Difficulty standing for long periods	7	2	3	1	–
Difficulty rolling over in bed	3	2	–	–	–
Difficulty walking for a long time	1	6	**4**	–	1
Difficulty walking inside the house	–	1	1	–	–
Difficulty walking outdoors	–	1	**5**	–	–
Difficulty walking in unfamiliar places	1	1	**6**	–	–
Difficulty walking in a crowded room or area	1	–	–	–	–
Difficulty with walking down the stairs	1	–	1	–	–
Difficulty with walking up the stairs	1	–	–	–	–
Dragging your leg or foot when walking	9	–	3	4	1
Needing to concentrate on your walking	9	–	3	3	1
Needing to put more effort on your walking	5	2	**5**	2	1
Problems with your arm swing when walking	17	–	1	2	–
Problems with your balance when standing still	4	1	2	2	–
Problems with your balance when walking	7	1	1	2	–
Problems with your balance when moving	2	2	**5**	1	–
Clumsiness when walking	2	–	–	1	–
Shuffling when walking	1	–	1	1	–
Sudden freezing when walking	–	1	2	2	–
Walking with a limp	2	3	–	2	–
Stumbling when walking	2	–	1	2	–
Falling	3	–	–	1	1

PRO: patient-reported outcome

*Group A, N = 30; Group B, N = 29. One transcript from Group A was unable to be analysed due to poor audio quality, resulting in 30, rather than 31, cognitive debriefing participants for this group

^†^No items were reported as being least relevant

^‡^Items with greatest clarity issues are marked in bold

## Discussion

The novel PRO instruments reported here aim to address the current need in clinical research for improved outcome measures to detect and potentially track or monitor functional changes in people living with early-stage Parkinson’s. They were specifically designed to assess functional aspects of daily living, covering the concepts most important to people living with early-stage Parkinson’s, and to potentially demonstrate meaningful treatment benefit in this context. Evidence from people living with Parkinson’s informed the item content, language, and response option structure. With a greater focus on clinical trials investigating disease-modifying treatments [33] (i.e., therapies aimed to delay/slow progression by addressing the underlying biology of Parkinson’s), we believe that the granular nature of the items and response option structure of the PRO instruments will improve researchers’ ability to assess small, but meaningful changes in outcomes over time and thus enhance the overall potential to detect the effects of emerging therapies developed to slow disease progression. This assumption now needs to be tested in future clinical studies.

Patient experts were key throughout the PRO instrument development process, playing an important collaborative role within the research team in many areas, including: drafting new PRO items; reviewing the interview guide for the cognitive debriefing, testing time to completion of the PROs; contributing to the interpretation of cognitive debriefing findings; and facilitating decisions on the final PRO content including refinement and cultural adaption of the items. They also provided considerations on future research needs; for example, investigating whether the dominant side or nondominant side impacts the overall scoring of disability for a person living with Parkinson’s. Final decisions were made once the multidisciplinary research group had reached a consensus, which ensured that the content of the novel PRO instruments was patient-centric, as well as viable and meaningful from a clinical, regulatory and measurement perspective.

Cognitive debriefing findings demonstrated that the newly generated PRO items were relevant to the experience of the cognitive debriefing participants and, generally, clear and easy to understand and respond to. No issues were identified with the instructions, likely due to the input of the patient experts in developing the instructions. The novel PRO instruments also fill the gaps present in existing PRO instruments by assessing functional slowness, mobility and fine motor skills, which were recognised to be of cardinal importance in early-stage Parkinson’s [19]. In phase 1, study participants reported that ‘slowness’, rather than ‘difficulty’ in completing tasks, activities, and functions, relates to not just motor but also cognitive demands [19]. It was also agreed that focusing on more subtle gait abnormalities is of particular importance to people living with early-stage Parkinson’s [19]. Therefore, items related to cognitive slowness concepts and subtle gait abnormalities were included, underscoring the relevance of the item content for the Early Parkinson’s Function Slowness and Mobility PRO measures in early-stage Parkinson’s and its potential for reliable, valid, and sensitive measurement in this setting.

Future research outcomes will inform the finalization of the item content in both PRO instruments, for which there is early evidence for potential item redundancies. Although the current item sets are lengthy, the multidisciplinary research group decided to defer final decisions on item reduction until more evidence is available, so that item reduction decisions could be based on both qualitative and quantitative evidence, especially considering that (i) the battery of items are short, and easily read and completed by people living with early-stage Parkinson’s; (ii) more items may warrant better measurement in terms of validity and reliability; and (iii) the draft PRO instruments require further testing in people living with early-stage Parkinson’s in order to inform final reduction decisions.

These novel PRO instruments are being tested in a phase 2 clinical study investigating the safety and tolerability of minzasolmin in people living with early-stage Parkinson’s (NCT04658186) [34] and in a 2-year observational study with de novo people living with Parkinson’s (NCT04985539) [35]. Additionally, qualitative research is ongoing in the USA, the Netherlands, and Asia in people with clinically confirmed Parkinson’s across various levels of disease severity to further ascertain the content validity of the PRO instruments, to eliminate questions that are vague, redundant and ambiguous, and to single out individual items reflective of the experience of living with early-stage Parkinson’s that evolve to become the symptoms creating disability evident in the later stages of Parkinson’s.

Although there is additional research being conducted, we consider that these novel PRO instruments may complement or represent viable alternatives to MDS-UPDRS part II for the assessment of functional aspects of daily living in people with early-stage Parkinson’s. The concurrent assessment of the PRO instruments and the MDS-UPDRS part II Activities of Daily Living Scale (currently ongoing) will bring about a more accurate and detailed portrait of the draft PRO instruments and their relationship to the progression of Parkinson’s disability over time, beginning with its earliest stages. The effect of symptomatic treatment on activities of daily living, as well as ageing as a confounder, will also be explored with these instruments. The large amount of qualitative evidence overseen by the multidisciplinary research group (including direct input by patient experts) in developing these instruments supports our assessment that the new Early Parkinson’s Function Slowness and Early Parkinson’s Mobility PRO instruments are appropriate and comprehensive, whilst potentially improving the ability to demonstrate meaningful treatment benefits in early-stage Parkinson’s daily functioning by evaluating concepts important to people with the condition.

A lack of quantitative data represents a current limitation of the study; however, mixed methods research is ongoing and full psychometric analyses are planned. Another limitation of this study is the lack of diversity of the study population; only participants from the UK and USA were interviewed, of which, 95% (57 out of 60) identified as White. As such, the results may not be generalizable to other ethnicities or the global community of people living with Parkinson’s. The research team is working on a shared diversity, equity, and inclusion strategy for future work. Furthermore, involvement with PAOs may lead to better informed patient experts; as such, including patient experts who are not involved with PAOs would provide additional perspectives in future PRO studies.

## Conclusion

The novel PRO instruments developed through this research have the potential to help fill the gaps of existing PRO instruments by assessing functional daily living in the context of early-stage Parkinson’s. The PRO instruments have been developed with direct input from patient experts and, separate to patient experts, extensive qualitative work was obtained from interviews with cognitive debriefing participants who were living with early-stage Parkinson’s (in addition to the 50 interviews with people living with early-stage Parkinson’s that were conducted in phase 1). As such, the PRO instruments provide strong evidence of content validity for assessing symptoms that are meaningful to people living with the early stages of the condition and represent clinically relevant outcome measures for use in clinical trials. Further evidence, review, and refinement of the item content are warranted to ensure optimal clinical- and patient-meaningfulness of the item set.

## Supplementary Information

**Additional file 1.** Supplementary Tables.

## Data Availability

Data from non-interventional studies are outside of UCB’s data sharing policy and are unavailable for sharing.

## Abbreviations

COA: Clinical outcome assessments
MDS: Movement Disorder Society
PAO: Patient advocacy organizations
PPMI: Parkinson’s Progression Markers Initiative
PDQ-39: 39-Item Parkinson’s disease questionnaire
PRO: Patient-reported outcome
UPDRS: Unified Parkinson’s Disease Rating Scale
UK: United Kingdom
USA: United States of America
