# Supplementary material for: Development and early qualitative evidence of two novel patient-reported outcome instruments to assess daily functioning in people with early-stage Parkinson’s
Source: J Patient Rep Outcomes. 2023 Apr 20;7:40. doi: 10.1186/s41687-023-00577-9 (PMC10119343; doi:10.1186/s41687-023-00577-9)
Supplement: Supplementary file 1 — Additional file 1. Supplementary Tables. [file 41687_2023_577_MOESM1_ESM.docx]

**Supplementary Table 1** Example questions used in the interviews

| **Examples of questions asked following spontaneous feedback:** | | | |
| --- | --- | --- | --- |
| *Instruct participants to read all PRO instrument questions out loud and ask them to discuss how they would respond to each of them. If participant reports or indirectly indicates any issues with the questions or response options, please ask the following suggested questions (depending on the reported issue):* | | | |
| - Clarity:   - How would you phrase it differently?   - How would you put the question in your own words? | | | |
| - Relevance:   - Has this symptom not been relevant for you over the past 7 days or recently, or has this never been relevant for you, i.e., have you never experienced this symptom? | | | |
| - Ease of completion:   - What response options would you use instead?     - This question can be asked if the participant reports having difficulty selecting one of the given response categories, or appears to indicate that by their thought process | | | |
| **Examples of the open-ended questions asked to reveal further information about the PRO instruments:** | | | |
| **Issue:** | **Early Parkinson’s Function Slowness PRO** | | **Early Parkinson’s Mobility PRO** |
| Clarity | **Instructions:** Were the participants clear with the instruction of this PRO instrument? If not, then you could ask what they understand by ‘slowness in performing daily activities’?  **Question 42 (‘Reacting to things in real time to avoid potential accidents’):** Ask participants to give their interpretation of this question, providing  examples if appropriate | |  |
|  | **Conceptual overlap:** Are there any questions that appear to be measuring the same concept (for example, ‘using a knife’ and ‘using a fork’)? | | |
| Relevance | **Relevance:** Are there any questions here not relevant to your experience of living with Parkinson’s? | | |
|  | **Most/least relevant:** Which are the most/least  relevant questions in relation to your own  experience of slowness in performing daily  activities? | **Most/least relevant:** Which are the most/least  relevant questions in relation to your experience of walking and moving problems? | |
| Missing Concepts | **Missing concepts:** Are there any types of activities that you experience slowness in that are not covered  by these questions? | **Missing concepts:** Are there any moving or walking problems you experience that are not covered by these questions? | |
| Final Feedback | **Final feedback:** Do you have any other feedback on this questionnaire? | | |

PRO: patient-reported outcome

**Supplementary Table 2** PRO item issues that arose from the CD interviews

| **PRO scale or item (version 0.3 as presented at the CD interviews)** | **Criteria where evidence indicated issues with the draft items** | |  |
| --- | --- | --- | --- |
|  | **Conceptual uniqueness** | **Item clarity/ quality** | |
| **Early Parkinson’s Function Slowness PRO** |  |  | |
| Doing up buttons | + | + | |
| Doing up a zip | + | + | |
| Using a knife | + |  | |
| Using a fork | + |  | |
| Counting money |  | + | |
| Carrying or moving things | + |  | |
| Lifting or carrying things | + | + | |
| Climbing up or down the stairs |  | + | |
| Walking on uneven ground | + |  | |
| Walking on a busy street or crowded area | + |  | |
| Doing your hobbies or other leisure activities |  | + | |
| Performing your work or tasks within your daily routine |  | + | |
| Multitasking |  | + | |
| Thinking or processing things | + |  | |
| Reacting to things in real time to avoid potential accidents |  | + | |
|  |  |  |  |
| **Early Parkinson’s Mobility** |  |  | |
| Difficulty getting out of a chair |  | + | |
| Difficulty walking outdoors | + | + | |
| Difficulty walking in unfamiliar places |  | + | |
| Difficulty bending |  | + | |
| Needing to put more effort on your walking | + | + | |
| Difficulty rolling over in bed |  | + | |
| Needing to concentrate on your walking | + |  | |
| Clumsiness when walking | + |  | |
| Shuffling when walking | + |  | |
| Problems with your balance when moving | + | + | |
| Problems with your balance when walking | + |  | |
| Difficulty standing for long periods |  | + | |
| Difficulty sitting for long periods |  | + | |
| Difficulty walking for a long time | + | + | |
| Problems with your arm swing when walking |  | + | |
| Sudden freezing when walking |  | + | |
| Falling |  | + | |

+, which criteria the indicated issue with the draft item corresponds to

CD: cognitive debriefing; PRO: patient-reported outcome
